# Curated occurrence records for native millipedes in Tasmania, Australia

**DOI:** 10.3897/BDJ.13.e165529

**Published:** 2025-08-12

**Authors:** Robert Evan Mesibov

**Affiliations:** 1 No affiliation for this profile, West Ulverstone, Tasmania, Australia No affiliation for this profile West Ulverstone, Tasmania Australia

**Keywords:** Australia, Tasmania, Diplopoda, Polyxenida, Polyzoniida, Sphaerotheriida, Spirostreptida, Chordeumatida, Polydesmida

## Abstract

**Background:**

Tasmania is the smallest of the eight continental Australian States and Territories, with a landmass of ca 68000 sq. km. In comparison to the rest of Australia, Tasmania's millipede fauna is relatively well-sampled and well-studied, and includes 135 formally described native species in five orders: Polyxenida (5 species), Sphaerotheriida (2), Spirostreptida (21), Chordeumatida (11) and Polydesmida (96). Another 84 species (Polyzoniida 7, Polydesmida 76, Chordeumatida 1) have been sorted to morphospecies but not yet described.

There is currently no complete, publicly available compilation of occurrence records for both described and undescribed Tasmanian millipedes. I privately maintain a dataset of this kind for generating distribution maps on the "Tasmanian Millipedes" website (https://www.datafix.com.au/tasmanian_millipedes/). In 2018 I shared more than 7000 records for named, native species in Tasmania with the Atlas of Living Australia (https://collections.ala.org.au/public/showDataResource/dr444), but the ALA resource has not been updated since 2019 and does not include either undescribed species occurrences or the large number of newer records for named species.

**New information:**

This paper describes a public version in Darwin Core format of 10073 curated occurrence records for native Tasmanian millipedes, and seven records for three Polydesmida species native to the Australian mainland which have been introduced to Tasmania. The dataset does not include records for introduced European Julida and Polydesmida. Some other exclusions are noted in the General description section, below.

## Introduction

Expert-curated occurrence datasets are valuable additions to the corpus of publicly available biodiversity data. Recent contributions include datasets for fungal occurrences in Australia (Hao et al. 2021), for European groundwater-dwelling copepods (Cerasoli et al. 2025) and for the insect order Zoraptera worldwide (Kaláb et al. 2025). Compiling and maintaining such datasets requires careful data review, but the results are more reliable than simple combinations of records from diverse sources, and are more likely to be up-to-date.

There is currently no complete, publicly available compilation of occurrence records for both described and undescribed Tasmanian millipedes. I privately maintain a dataset of this kind for generating distribution maps on the "Tasmanian Millipedes" website (https://www.datafix.com.au/tasmanian_millipedes/). In 2018 I shared more than 7000 records for named, native species in Tasmania with the Atlas of Living Australia (https://collections.ala.org.au/public/showDataResource/dr444), but the ALA resource has not been updated since 2019 and does not include either undescribed species occurrences or the large number of newer records for named species. ALA later shared the dataset with GBIF (https://www.gbif.org/dataset/834442e7-c3ba-4ff9-bd1f-3288989f7725).

Here I describe a curated dataset of occurrence records for native Australian millipedes in Tasmania, based on museum specimen lots. The dataset includes described and undescribed species, and samples identified only to genus, family or order. It is intended to replace the ALA dataset and will be more frequently updated.

## General description

### Purpose

The Tasmanian millipedes dataset began as a component of a larger effort aimed at documenting occurrences for all native Australian millipedes. As described in Mesibov (2013), occurrence records were compiled and edited with the help of museum collection managers and millipede collectors, then made publicly available (as TSV and KML files) on the privately maintained *Millipedes of Australia* website. The website was retired in 2019 (Mesibov 2019) and users were referred to ALA and GBIF for records of named Australian millipedes.

In 2021 I established a website for the millipede fauna of Tasmania, *Tasmanian Millipedes*, again privately maintained and hosted on my own domain. To build distribution maps for the website I revised and expanded the relevant sections of the *Millipedes of Australia* records dataset. I added records for undescribed species and for millipedes only identified to genus, family or order.

All 10080 records in version 1 of the current dataset (Mesibov 2025) are for museum specimen lots, but the location of 39 of these lots is uncertain. There are no image-based millipede records, for example from *iNaturalist*.

The records in the dataset are "curated" in the sense that I have confidence in the identity and collection details of the 10080 specimen lots. I identified 97% of the lots myself, and almost all the remainder were identified by original describers, specialists or experienced Tasmanian collectors. About 64% of the lots are my own or joint collections, with collection details to 2020 curated as documented in Mesibov (2021), or added since I resumed field work in 2022. Collection details for the other 36% of the lots were checked as described in Mesibov (2013) or through correspondence with individual collectors.

Curation of the records has involved correcting errors in museum collection databases. In most cases the corrections have been accepted by collection managers (Mesibov 2013; K. Moore, Tasmanian Museum and Art Gallery, Hobart, pers. comm.). Erroneous records persist in museum databases, however, and have been shared with ALA, which in turn has shared them with GBIF. An important goal in publishing this dataset is to make corrected records publicly available.

Between 50 and 100 new Tasmanian millipede occurrence records are added each year to my privately maintained dataset. These new records will be added periodically to the curated dataset in the IPT, and more promptly to the distribution maps on the *Tasmanian Millipedes* website. I would be grateful to users of the published dataset for corrections and additions.

## Geographic coverage

### Description

Tasmania and its outlying islands

### Coordinates

-44 and -39 Latitude; 143 and 149 Longitude.

## Temporal coverage

### Notes

ca 1843 to 2025-06-27

## Usage licence

### Usage licence

Creative Commons Public Domain Waiver (CC-Zero)

## Data resources

### Data package title

Curated Darwin Core occurrence records for native millipedes in Tasmania, Australia

### Resource link

https://ipt.pensoft.net/resource?r=tasmilrecs-2025-07-06

### Number of data sets

1

### Data set 1.

#### Data set name

Occurrence records for native millipedes in Tasmania, Australia, curated by Robert Mesibov

#### Data format

Darwin Core archive (https://dwc.tdwg.org/terms/)

#### Character set

UTF-8

#### Download URL

https://www.gbif.org/dataset/9b37920c-8d47-41a0-8f90-064b792b6a15

#### Description

Version 1 of the dataset (to 2025-07-06) contains 10073 occurrence records for native Tasmanian millipedes and seven records for native Australian millipedes introduced to Tasmania. The records in the dataset are "curated" in the sense that I have confidence in the identity and collection details of the 10080 specimen lots. I identified 97% of the lots myself, and almost all the remainder were identified by original describers, specialists or experienced Tasmanian collectors. About 64% of the lots are my own or joint collections, with collection details to 2020 curated as documented in Mesibov (2021) or added since I resumed field work in 2022. Collection details for the other 36% of the lots were checked as described in Mesibov (2013) or through correspondence with individual collectors.

**Data set 1.** 

Column label	Column description
occurrenceID	A unique code for each occurrence
kingdom	Kingdom of the taxon; all "Animalia"
phylum	Phylum of the taxon; all "Arthropoda"
class	Class of the taxon; all "Diplopoda"
order	Order of the taxon
family	Family of the taxon, or "incertae sedis" if the taxon has not yet been assigned to a family
genus	Genus of the taxon
specificEpithet	Species name of the taxon
scientificName	Full scientific name of the taxon, including authorship for genus- and species-level taxa
scientificNameID	LSID for the taxon name in MilliBase (https://www.millibase.org/)
verbatimIdentification	Taxon name string; for described taxa, the same as the scientificName entry but without authorship; for undescribed taxa, a unique taxon code
taxonRank	Rank of the entry in scientificName; for undescribed taxa, the lowest assignable rank
basisOfRecord	Basis of the occurrence record; all "PreservedSpecimen"
institutionCode	Full name of the institution in which the specimen lot is deposited; blank for 39 specimen lots whose location is unknown
catalogNumber	Currently known museum registration number of specimen lot
individualCount	Number of specimens if known
organismRemarks	Abbreviated gender and life stage of specimens if known, e.g. "2M,2F,1J" for "2 males, 2 females, 1 juvenile"
occurrenceStatus	Status of occurrence at the given location; all "present"
occurrenceRemarks	Notes on taxon occurrence at the given location
typeStatus	Formal type status of specimen lot, e.g. "holotype of Bromodesmus rufus Mesibov, 2004"
taxonRemarks	Notes on the status of the taxon; either "possibly a species group", "suborder Dalodesmidea, family uncertain" or blank
identifiedBy	Name of the identifier(s)
identifiedByID	If available, personal ID of the identifier(s), either from the ORCID database or Wikidata
identificationRemarks	Notes on the identification
preparations	Preservation method
disposition	Notes on specimen lots or their registration numbers in collections, e.g. "formerly QVM 23:12180"
establishmentMeans	Status of the taxon in Tasmania; all "native" except "introduced" for three Paradoxosomatidae species native to Australia and now established in Tasmania
eventID	A unique code for each sampling event
country	Country for the event; all "Australia"
countryCode	Country code for the event; all "AU"
stateProvince	Australian state for the event; all "Tasmania"
locality	Normalised location string for the event
decimalLatitude	Latitude in Tasmania for the event, to four decimal places
decimalLongitude	Longitude in Tasmania for the event, to four decimal places
coordinateUncertaintyInMeters	Radius of a circle around the latitude/longitude point within which the event occurred
geodeticDatum	Datum for the latitude/longitude point; all "WGS84"
coordinatePrecision	Precision of the latitude/longitude estimates; all "0.0001"
verbatimCoordinates	Original coordinates recorded for the event, if known
verbatimSRS	Datum for the original coordinates for the event, if known
verbatimCoordinateSystem	Format system for the original coordinates, e.g. "UTM"
georeferenceSources	Source of the original coordinates, if known
georeferencedBy	Name of the person(s) providing the original coordinates, if known
minimumElevationInMeters	Estimate of minimum elevation above sea level for the event, mainly taken from LISTmap (Land Information Services Tasmania online mapper; https://maps.thelist.tas.gov.au/listmap/app/list/map)
maximumElevationInMeters	Estimate of maximum elevation above sea level for the event, mainly taken from LISTmap (Land Information Services Tasmania online mapper; https://maps.thelist.tas.gov.au/listmap/app/list/map)
eventDate	Date or interval date for an event in ISO 8601 format
recordedBy	Name of the specimen collector(s)
recordedByID	If available, personal ID of the specimen collector(s), either from the ORCID database or Wikidata
eventRemarks	Notes on the event, e.g date remarks or brief description of habitat
locationRemarks	Notes on the location of the event, e.g. plot codes or location ambiguities
samplingProtocol	Specimen collection method, if known
fieldNumber	Field or specimen lot code, other than museum registration number
footprintWKT	LINESTRING entries
footprintSRS	WKT datum; "EPSG:4326"
modified	Day in ISO 8601 format on which the record was created or last edited
georeferenceRemarks	Notes on the determination of the original coordinates

## Additional information

There are 8962 unique taxon occurrences in version 1 of the dataset, i.e. with unique identification, latitude/longitude and collection date. Of the 10080 total millipede occurrences, 8154 are identified to described species, 599 to morphospecies, 1173 to genus, 110 to family and 44 to order. Morphospecies are listed by code name in the *verbatimIdentification* field, but are assigned to a higher taxon in *scientificName* and *taxonRank*.
